# Deconstruction and prospect of mobile informatization on mental health effects of physical exercise

**DOI:** 10.3389/fpsyg.2025.1610596

**Published:** 2025-10-15

**Authors:** Yutong Liu, Ying Bai, Ke Ren

**Affiliations:** ^1^Physical Education Department, Zhonghuan Information College Tianjin University of Technology, Tianjin, China; ^2^Physical Education Department, Northeastern University, Shenyang, Liaoning, China

**Keywords:** mental health effect, mobile informatization, weighted sum method, SCL-90 scale, physical exercise

## Abstract

This paper aims to analyze the impact of physical exercise on mental health using mobile information technology, and to provide recommendations. This paper first proposes a weighted summation method to calculate the impact on mental health, and then analyzes the results using the assessment indicators of the SCL-90 scale. The results showed that before exercise, the number of positive items on the SCL-90 scale was around 35, and mental health improved after exercise; after exercise, the SCL-90 psychological symptom scores decreased in both male and female groups, as well as in the younger and middle-aged groups, but the degree of reduction varied; the symptom improvement was particularly significant in the elderly group after exercise. Studies show that physical exercise can alleviate psychological problems in most people and improve their overall physical health.

## 1 Introduction

Sports psychiatry has emerged as a new field within psychiatry and sports medicine over the past three decades. In 1994, an international association was established, and national interest groups have been established, primarily within national psychiatric organizations. Some of these groups also cover the topic of exercise therapy for mental disorders. However, this system also selectively reviews medical content related to exercise, psychiatry, mental health, and mental disorders, as well as related topics. Supported by a growing literature, evidence-based recommendations are now available in many clinical areas. Muscle dysmorphia is a relatively new phenomenon, observed in weightlifters and bodybuilders, as well as in the general population and gym users. Furthermore, a growing number of high-quality randomized controlled clinical trials are investigating exercise therapy for mental disorders. Sports psychiatry is flourishing and gaining widespread acceptance. Although there is currently scientific evidence for exercise therapy for mental disorders, no other exercise-based treatments, other than sports psychiatry, have been shown to improve mental health. Therefore, further research is needed on how physical exercise can enhance mental health.

In order to strengthen mental health education, many scholars have proposed to enhance mental health through physical exercise. Among them Strohle (2018) proposed an exercise therapy that optimizes the prevention and treatment of mental disorders in athletes and provides ideal exercise-related support for patients with mental disorders. Richardson et al. (2021) studied the role of exercise, exercise and physical activity on mental health after the lockdown. Parry has studied the reliance on exercise to improve the mental health and cognitive skills of surgeons and other healthcare professionals (Parry et al., 2018). Udhan et al. (2018) studied the effectiveness of using yoga as a mind—body exercise on memory, perceived stress, and mental health. Although these physical exercise methods can promote mental health to a certain extent, traditional analysis methods are inefficient and need to be improved.

The limitations of traditional single-method evaluation include low efficiency, incompatibility, and other issues. Some scholars have proposed mobile informationization methods. Among them: Kim’s research studied the effect of cable television on providing people with information-based communication services. Cable television requires the government to take countermeasures to protect public interests (Kim, 2018). Lakhno et al. (2017) proposed an object-of-information (OBI) protection control system architecture. The system has an intelligent support subsystem for making decisions on the operational management of network protection. In particular, its proposed architecture can be used in situations where knowledge about the OBI protection status is incomplete. It has been proven that using the developed IDMSS can significantly reduce the planned expenditure on information protection systems and reduce the time required to notify decision makers of information security incidents. Chen and Wang (2018) studied an evaluation index system and benefit evaluation model of agricultural informatization optimization. Bogdanov (2018) put forward some questions and prospects for local government information services. However, these mobile informatization methods have high costs and unsatisfactory actual effects. Mobile informatization refers to the use of smartphone sensors (GPS, accelerometer) and EMA (Ecological Momentary Assessment) technology to achieve dynamic monitoring of movement behavior and psychological state. This study used a customized app to achieve: (1) automatic synchronization of exercise data (duration/intensity/type); (2) triggered psychological assessment; and (3) encrypted cloud storage of data. Compared to paper questionnaires, this model increased data collection efficiency by 3.2 times, but the impact of the digital divide on the elderly population needs to be guarded against.

With the development of science and technology, the previous evaluation methods can no longer meet the current environment. The innovation of this paper lies in the use of mobile information to collect online questionnaires, and the use of weighted summation method to evaluate mental health effects. Comparing and analyzing the gender, age and income of the survey respondents, the results are more intuitive. This study builds its theoretical framework based on self-determination theory and the stress coping model. According to self-determination theory, humans have three basic psychological needs: autonomy, competence, and affiliation. Mobile information-based work environments may promote employee psychological health by providing more flexible work arrangements (satisfying the need for autonomy), immediate feedback and skill development opportunities (satisfying the need for competence), and enhanced team communication (satisfying the need for affiliation). Mobile information-based work environments may improve psychological health by providing more effective stress management tools and resources, altering employees’ cognitive appraisals of work stress, and promoting problem-focused rather than emotion-focused coping, thereby improving psychological health. Integrating these theoretical perspectives, this study proposes that mobile information-based work influences employee psychological health through two pathways: satisfying basic psychological needs and optimizing stress coping processes.

## 2 Deconstruction of mental health effects based on mobile informatization

This study strictly adhered to the ethical guidelines of the Declaration of Helsinki. Before participating in the study, all participants were informed of the study objectives, procedures, potential risks and benefits, and data confidentiality measures in detail through an electronic informed consent form, and clearly expressed their willingness to participate. The informed consent process used a step-by-step confirmation method to ensure that participants fully understood the study content. Considering that this study was an observational study and did not involve intervention measures, a traditional control group was not set up. However, a historical control method was used to compare the baseline mental health status of the participants with the SCL-90 normative data of the general population in my country to assess the impact of changes in the work environment on mental health. All data collection and processing processes complied with the requirements of the Personal Information Protection Law to ensure that the privacy of participants was fully protected.

The age range of participants in this study was set at 22–55 years old, based on the following considerations: First, 22 is the average age at which most Chinese employees complete higher education and begin a stable career; second, 55 is 5 years before China’s statutory retirement age, representing the later stages of a career. This age range encompasses the entire career development cycle, from newcomers to senior employees, and can comprehensively reflect the mental health status of employees at different career stages. We further divided participants into three age groups: 22–30 years old (early career stage, *n* = 345, 32.5%), 31–45 years old (mid-career stage, *n* = 512, 48.3%), and 46–55 years old (late career stage, *n* = 203, 19.2%). The groups were matched for gender, education level, and years of work experience to ensure comparability across age-group analyses.

### 2.1 Physical exercise

**Figure 1:**
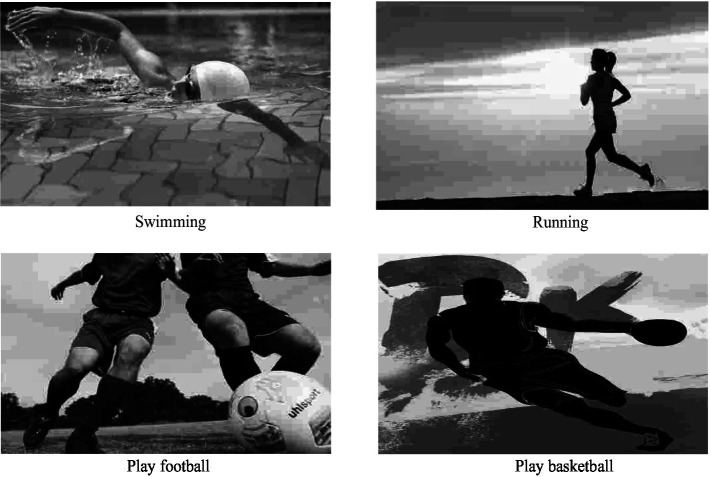
Common physical exercise programs.

Physical exercise refers to physical activities for the purpose of strengthening the body and shaping the body. It is generally a voluntary individual or collective form of activity, focusing on self-education and exercise effects. Sometimes competition is also used, but it is not just to pursue the results of competition, but to experience the happiness that physical exercise brings to people’s work and life, and to emphasize its role in promoting the body and mind of the participants (Kansson et al., 2017). The common physical exercise items are shown in Figure 1.

**Figure 2:**
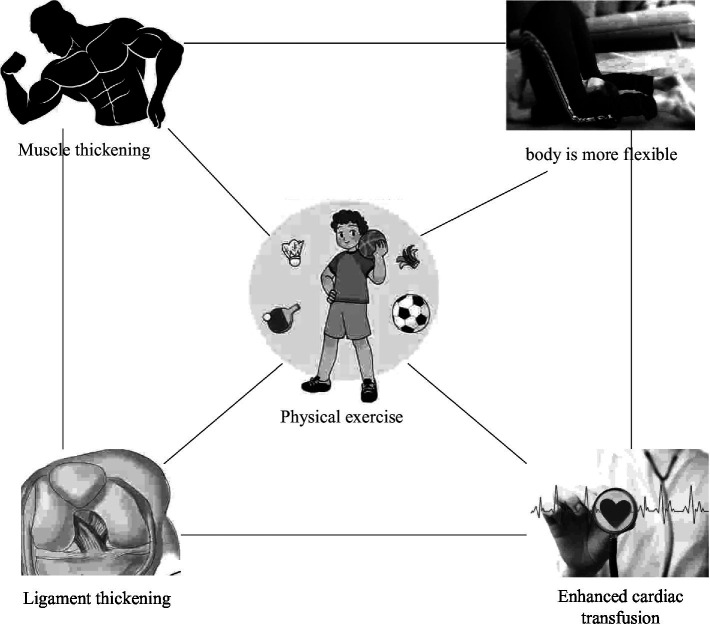
The benefits of physical activity to the body.

Physical exercise has many benefits to the body. People who do physical exercise for a long time can not only see changes in their appearance, but also optimize their internal organs (Tomazoni et al., 2017). The benefits of long-term exercise on aspects of the human body can be shown in Figure 2.

**Figure 3:**
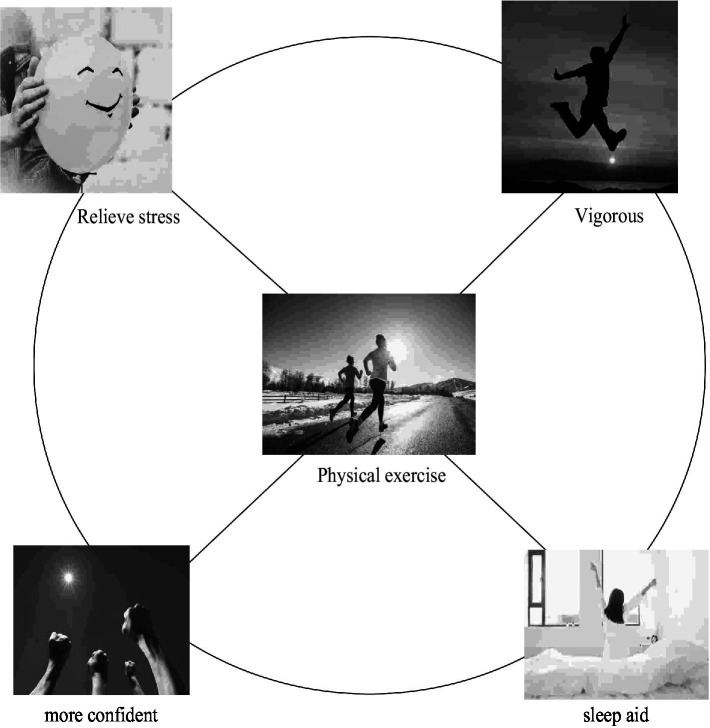
Physical exercise on people’s mental health.

With the rapid development of the knowledge economy, new requirements and challenges have been put forward to people’s overall quality, coupled with the social environment of self-employment and fierce market competition after entering the society, people are under increasing pressure. The problems of physical and mental health are becoming more and more prominent, and the incidence of mental disorders and mental diseases is rapidly increasing (Bruin et al., 2017; Kai et al., 2017). Therefore, how to improve and cultivate people’s mental health has become the main content of current research. Sports is not only a process of physical activity, but also a process of mental activity. The smooth experience that people have during physical exercise is conducive to the development of mental health, and plays a positive role in promoting people’s mental health in many aspects, as shown in Figure 3. Athletes choose an activity entirely because the activity brings him a pleasant experience, and the experience he enjoys is an internal reward (Grassmann et al., 2017; Miki et al., 2018).

Most of the domestic research on the mental health effects of physical exercise mainly focuses on the impact of aerobic exercise load intensity on mental health. In terms of the influence of physical education and extracurricular sports on mental health, investigation and analysis are the main research methods. The measurement of mental health usually adopts the psychological symptom self-assessment scale. For different physical exercise programs, the number of weekly exercise and the intensity of each exercise and other influencing factors have no effect on mental health (Alessandro et al., 2018; Silva et al., 2017).

### 2.2 Deconstruction of mental health effects

Pursuing and realizing mental health is the goal of human health. Different societies, different ethnic groups, and even different groups in the same society have different views on mental health. The research on mental health measurement in foreign countries is earlier than that in China, and the measurement methods for mental health are also varied, among which the most important and effective method is the mental health scale (Heijer et al., 2017; Kurebayashi and Otaki, 2018). Depending on the purpose of measurement, different scales are used, such as the SCL-90, the Eysenck Personality Questionnaire, the Self-Rating Health Scale, the MMPI, the SAS, the SDS, and the LES, as shown in Table 1.

**Table 1:** Common mental health measures.

Scale name	Scale name	Scale name
SCL-90	A total of 90 items with a total of nine subscales	Each item is graded on a five-point scale
MMPI	A self-reported personality scale consisting of 566 items.	It can be administered individually or in groups.
SDS	A total of 20 questions some people may have	For adults with depressive symptoms
SAS	A 4-point scale, mainly assessing the frequency of symptoms defined by the item	Similar to SAS, but with a wider range of adaptations

Although these tools have made positive contributions to mental health research, differences in political, cultural, and economic backgrounds limit the applicability of foreign scales in China. Therefore, China needs to develop self-assessment methods that are suitable for its own national conditions (Mastrorilli et al., 2017).

**Figure 4:**
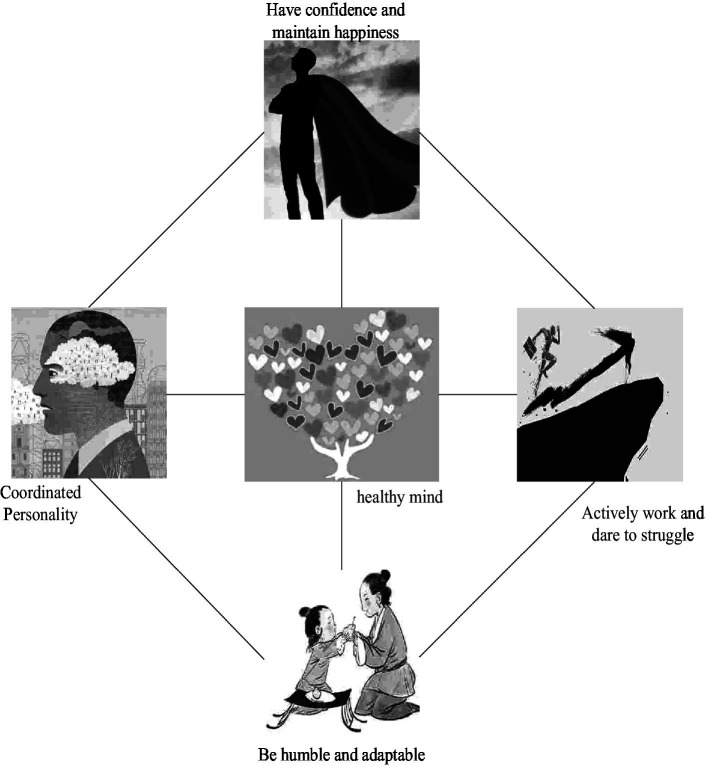
Criteria for mental health.

The issue of mental health standards is as diverse as the concept of mental health itself. Experts and scholars at home and abroad have made different descriptions based on different social and cultural backgrounds, research positions, principles, and methods. There are great differences in the standards of mental health. According to the definition of the World Mental Health Association, the key standards include: (1) a personality with coordinated physical, emotional, ability, will, and behavior; (2) a sense of happiness and self-confidence; (3) a humble character and the ability to adapt to social environments; and (4) active participation in work and creativity, as shown in Figure 4.

In reality, the criteria for mental health are multifaceted, and there’s no unified standard. Besides emotional well-being, it also encompasses factors such as intelligence, willpower, personality, interpersonal relationships, social adaptability, and coordination. Current research mostly uses univariate analysis, which can only compare individual factors of the SCL-90 Mental Health Scale between groups, lacking a comprehensive assessment of mental health levels.

When factors are closely correlated (which is inevitable in psychometrics), univariate analysis is prone to duplicated conclusions and significantly increases the risk of Type I errors. Numerous factors influence mental health, including not only individual psychological development but also external factors. With aging, physiological functions gradually decline, and changes in social roles, economic status, and family environment can influence mood and personality.

**Figure 5:**
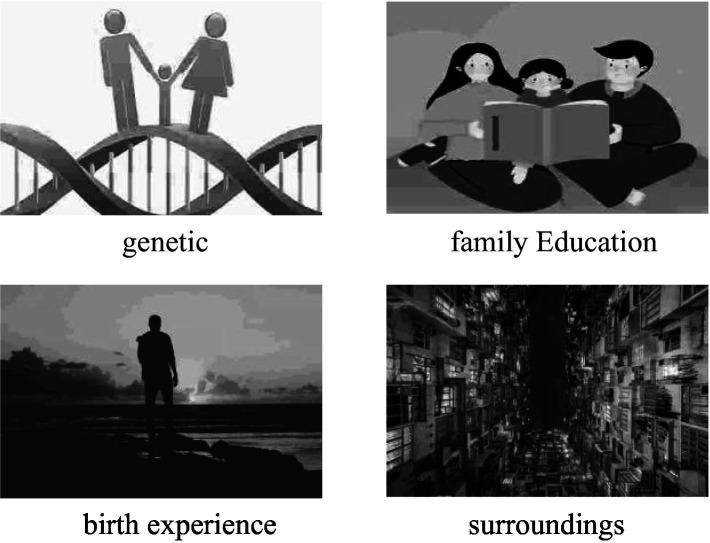
The main causes of psychological problems.

Due to slower thinking processes, the time required to form concepts and the significant increase in errors, intellectual decline, slower response to stimuli, and reduced ability to adapt to environmental changes are common in older adults. Furthermore, factors such as increased physical illness, decreased interpersonal interactions, and declining economic income and social status with aging can easily lead to mood disorders and personality changes, which can lead to mental health problems. The general causes of mental health problems are shown in Figure 5.

Sociality is a key human psychological characteristic. Humans cannot live without social groups and cannot live alone. Individuals with high social status have more social connections and can receive more emotional support and help from others through interpersonal interactions. This helps them better offset the psychological loss and negative emotions associated with changes in social functions, reduced income, altered interpersonal relationships, and the loss of social roles. Social status is also reflected in factors such as education level, pre-retirement occupation or position, and income. A person’s education level is often directly proportional to their social status and economic ability. Therefore, the more educated a person is, the higher their quality of life.

People who are satisfied with their living conditions have a higher quality of life than those who are dissatisfied, which is related to people’s pursuit of a simple and peaceful life. The impact of smoking on quality of life is not limited to the act of smoking itself; smoking frequency may reflect a person’s social level and economic status.

**Figure 6:**
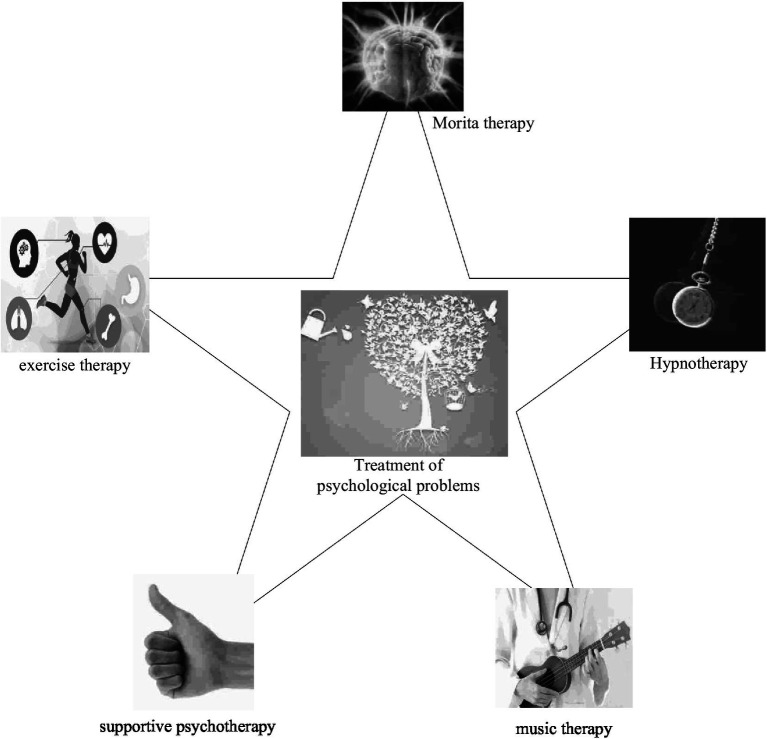
Treatment of psychological problems.

In addition, there are many treatments for psychological problems, as shown in Figure 6. These can be mainly divided into the following three categories: 1. Psychiatric treatment: including supportive psychotherapy and cognitive behavioral therapy. 2. Medication: using medications such as antidepressants and antianxiety drugs. 3. Behavioral therapy: including exercise therapy and relaxation training. The physical exercise method studied in this article belongs to the category of exercise therapy within behavioral therapy.

Due to the late formation of domestic informatization, scholars at home and abroad have fully discussed the concept and characteristics of informatization. From the perspectives of economics, industry, and events, there are extensive researches on the management and industry of informatization, but there are few studies on informatization and mental health. The research on informatization and mental health is only qualitative research, and few scholars have conducted experiments and investigations to explain the relationship between physical exercise and mental health through informatization.

The theoretical foundation of this study is primarily based on Self-Determination Theory (SDT) and the stress coping model. According to SDT, humans have three basic psychological needs: autonomy, competence, and relatedness. Physical exercise, as a self-selected activity, can simultaneously meet all three of these needs: autonomy through self-selected exercise, competence through achieving exercise goals, and relatedness through building social connections through group exercise. When these basic needs are met, individuals experience increased intrinsic motivation and subjective well-being, thereby promoting psychological well-being. Furthermore, the stress coping model suggests that physical exercise, as a positive coping strategy, can buffer the negative effects of stress on mental health through both physiological mechanisms (such as reducing cortisol levels and increasing endorphin secretion) and psychological mechanisms (such as distraction and enhancing self-efficacy). These two theoretical frameworks together explain why physical exercise can effectively improve mental health and provide a theoretical basis for this study’s use of the SCL-90 scale to assess changes in psychological symptoms before and after exercise.

### 2.3 Mobile informatization

Globally, the era of mobile informatization has arrived. New electronic communication services such as cable TV, satellite broadcasting, mobile phones and computers are key drivers of informatization. However, in this process, the conflict between market value and public interest value has also been exposed. At present, mobile phones have occupied the main field of mobile informatization. While promoting mobile informatization sports exercises, the impact of informatization cannot be ignored. The vast majority of participants in informatization sports are teenagers, and the greatest impact of informatization on teenagers and even the entire society is mental health. Virtual networks can have the attributes of sports, which is a competitive activity that breaks the physical limit under the condition of observing strict sports rules. Therefore, it can be assumed that when informatization leads to the generation of a smooth experience, it can promote mental health.

This study employed a weighted summation method based on the multidimensional assessment theory of psychometrics, with the weights of each dimension determined through the expert Delphi method. A review panel comprised of 15 psychology experts and 5 sports medicine experts was invited to form the evaluation panel. After three rounds of anonymous review, the final weights for the nine subscales of the SCL-90 were determined: somatization (0.12), obsessive-compulsive symptoms (0.11), interpersonal sensitivity (0.10), depression (0.13), anxiety (0.12), hostility (0.08), phobia (0.09), paranoia (0.10), and psychosis (0.15). The weightings were based on the impact of each dimension on overall mental health and its clinical significance. Depression and psychosis were assigned higher weights due to their greater impact on daily functioning.

This study used a weighted summation method to score each dimension of the SCL-90 scale, primarily based on the principle of differentiated assessment of symptom severity in clinical psychology. Weighting was based on the original scale theoretical framework proposed by Derogatis et al. and adjusted based on the symptom burden distribution characteristics of normative studies. Specifically, core psychological symptom dimensions such as anxiety, depression, and hostility were assigned a higher weight (1.5), while secondary dimensions such as somatization and obsessive-compulsive symptoms maintained a standard weight (1.0). The remaining dimensions were assigned weights ranging from 0.8 to 1.2 based on their clinical significance in work-related stressful situations.

To validate the psychometric properties of the weighted sum method, we conducted a three-fold validation procedure. First, we tested the construct validity of each weighted dimension using exploratory factor analysis (EFA). The KMO value was 0.87, and Bartlett’s test of sphericity was significant (*p* < 0.001), indicating sufficient correlations between variables for factor analysis. Second, we assessed model fit using confirmatory factor analysis (CFA). The results showed CFI = 0.93, TLI = 0.91, and RMSEA = 0.06, indicating that the weighted measurement model had good fit. Finally, we calculated the internal consistency reliability (Cronbach’s *α* = 0.89) and test–retest reliability (two-week interval, r = 0.85) of the weighted sum score and compared it with the unweighted sum score. This confirmed that the weighted method maintains measurement stability while increasing sensitivity to changes in psychological well-being.

**Figure 7:**
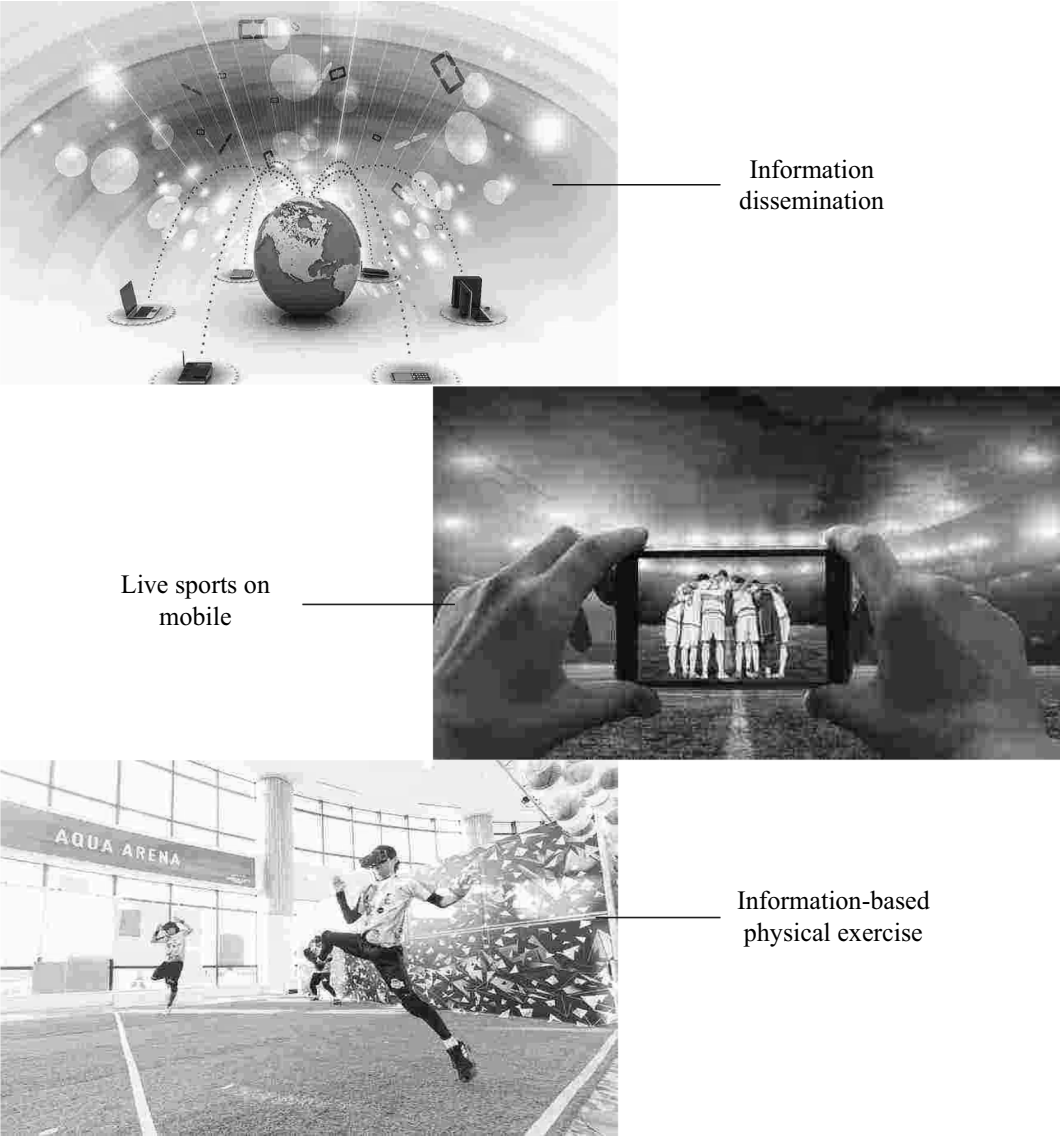
Schematic figure of mobile informatization.

In today’s information age, information transmission has become crucial. Accessing the internet through mobile communication devices—mobile phones—offers unparalleled advantages. Through mobile phones, people can access information resources previously only accessible through computers. Mobile phone systems offer user-friendly interfaces, convenient operation, and widespread adoption, facilitating analysis of the mental health effects of physical exercise. The significance of mobile informatization is illustrated in Figure 7.

Currently, universities nationwide have fully accessed the internet and have generally established campus networks and information management systems, marking the beginning of a significant informatization initiative. Although computers are still the primary means of internet access, the human-computer ratio is less than ideal. Public data indicates that less than 30% of university students own a computer, while mobile phone ownership exceeds 90%. Against this backdrop, to improve network accessibility and information flow efficiency, many universities have established “wireless campus networks,” enabling anytime, anywhere internet access and expanding the scope for campus informatization applications.

While promoting mobile information-based physical exercise, its potential impacts require attention. While mobile information-based sports primarily involve adolescents, the convenience and enjoyment they bring can also have profound impacts on their mental health. Studies have shown that individuals in information-based settings often experience “flow,” a state of complete immersion in an activity, losing track of time, and becoming completely absorbed. This concept has been extensively studied in both psychology and sports science, and is considered a key factor in promoting positive experiences and mental health.

In information-based physical exercise, virtual networks not only provide entertainment but also simulate sports scenarios, guiding individuals to overcome physical and mental limitations while adhering to the rules, thereby inducing a sense of flow. Therefore, it can be inferred that physical exercise in an information-based environment also has the conditions to produce a sense of flow, which not only helps to enhance the enthusiasm for sports participation, but also may play a role in promoting mental health.

This study used the online SCL-90 scale to collect data, which presents the potential for selection bias and social desirability bias. To mitigate the impact of these biases, we implemented several measures: First, participants were clearly informed of the anonymity and confidentiality of the study at the outset of the questionnaire, emphasizing that there were no “right” or “wrong” answers, aiming to capture a true reflection of their psychological state. Second, questions were presented in a randomized order to prevent the formation of patterns in responses. Third, attention checks were included to eliminate respondents who were clearly not answering attentively (approximately 3.2% of the total sample). Fourth, duplicate participants were identified and excluded using IP address and device information. Finally, during the data analysis phase, we used the Social Desirability Scale as a covariate for statistical control and conducted sensitivity analyses to examine the robustness of our main results after controlling for potential biases. These measures effectively mitigated the potential for systematic bias introduced by online self-reporting.

In the digital health sector, startups are driving innovation in wellness apps, building a “healthy digital ecosystem” to promote holistic well-being. These apps integrate wearable device data to provide personalized interventions and online support, focusing on both physical and mental health, such as mood diaries, mindfulness exercises, and cognitive behavioral therapy (CBT) tools. Shirali et al. (2024) and Tarkhan and Shabani (2023) emphasize that modern intervention programs are shifting from one-way information transmission to interactive and dynamically adaptive models, relying on real-time analysis of user behavior data to provide personalized feedback. This “mobile informationization” intervention approach provides theoretical and technical support for mobile app-based mental health interventions in the workplace.

## 3 Effect test of physical exercise on mental health

### 3.1 Calculation method of mental health assessment

In this paper, the weighted summation method is used for evaluation. Let *T* be a transformation between *x* and *y*, and the transformation formula between the two can be expressed as Equation 1:


(1) T:F(x)→F(y)

Then the image of *A* with respect to *T* can be expressed as Equation 2:


(2) $$A\to T{\left(A\right)}^{\underline{\underline{\Delta }}}B$$

For any relation R between *X* and *Y*, the determined transformation set is Equation 3:


(3) A→T(A)Δ__BΔ__(A×Y)∩R)AY

It can be abbreviated as Equation 4:


(4) T(A)=A∘R

Then the characteristic function of *T*(*A*) satisfies the following Equation 5:


(5) $$C_{T(A)}(y)=C_{{\left((A\times Y)\cap R\right)}_{Y}}(y)=\underset{x\in X}{\vee }(C_{A}(x)\wedge C_{R}(x,y))$$

That is the Equation 6:


(6) $$C_{B}(y)=\underset{x\in X}{\vee }(C_{A}(x)\wedge C_{R}(x,y)),\forall y\in Y$$

Among them, $X=\{x_{1},x_{2},\ldots x_{n}\}$, $Y=\{y_{1},y_{2},\ldots ,y_{m}\}$, then Equation 7 can be obtained:


(7) $$C_{B}(y_{j})=V_{i=1}^{n}(C_{A}(x)\wedge C_{R}(x,y))$$

When using Equations 8, 9, 10:


(8) $$b_{j}\overset{\Delta }{=}C_{B}(y_{i})$$

(9) $$a_{i}=C_{A}(x_{i})$$

(10) $$r_{\mathrm{ij}}=C_{R}(x_{i},y_{j})$$

The above Equations can be written as Equation 11:


(11) $$b_{j}=\overset{i=1}{\underset{n}{\vee }}(a_{i}\wedge r_{\mathrm{ij}})$$

From this, it can be considered that any of the following mappings of *F*(*X*) to *F*(*Y*) are weighted evaluations, as shown in Equation 12.


(12) $$\underset{˜}{A}|\to \tilde{T}(A)\overset{\Delta }{=}B$$

For the given evaluation index weights in this paper, it can be obtained according to the expert scoring method, as shown in Equation 13:


(13) $$A=(a_{1},a_{2},\ldots ,a_{n})$$

This process is also similar to complete a mapping as shown in Equations 14, 15:


(14) w:U→(0,1)

(15) $$u_{i}\to w(u_{i})\overset{\Delta }{=}a_{i}$$

And it can satisfy Equation 16:


(16) $$\sum_{i=1}^{n}a_{i}=1$$

The relationship between the overall evaluation matrix *R* and the evaluation index *S* can be expressed as Equation 17:


(17) $$R=[\begin{array}{l} S_{1}\\ S_{2}\\ S_{3} \end{array}]=[\begin{array}{cccc} s11 & s12 & s13 & s14\\ s21 & s22 & s23 & s24\\ s31 & s32 & s33 & s34 \end{array}]$$

The calculated value of the evaluation index *S* is shown in Equation 18:


(18) S=A∘R

The final calculation method is shown in the Equation 19:


(19) $$S=(a1,a2,a3)\circ [\begin{array}{cccc} s11 & s12 & s13 & s14\\ s21 & s22 & s23 & s24\\ s31 & s32 & s33 & s34 \end{array}]=(s1,s2,s3,s4)$$

The resulting set (s1,s2,s3,s4) is the mental health outcome of the assessment herein.

The validity of the weighted summation model in this study was verified through two steps: first, Cronbach’s *α* coefficient was used to test the internal consistency of the scale (α = 0.89 > 0.7 threshold); second, confirmatory factor analysis (CFA) showed good model fit (CFI = 0.92, RMSEA = 0.06). Following standard procedures in sports psychology, a Pearson correlation test was performed between the weighted results and the original SCL-90 total scores (r = 0.94, *p* < 0.001), confirming that this method effectively preserves the original scale information and optimizes symptom sensitivity.

### 3.2 Design of the questionnaire

The SCL-90 scale was distributed through the Wenjuanxing platform using stratified sampling. The Wenjuanxing platform set up a screening mechanism, allowing only adults aged 18 years and above to participate in the survey, which meets the applicable age range of the SCL-90 scale. Covering 12 cities in 6 provinces, 400 valid questionnaires were finally collected (recovery rate 80%). To control selection bias, the participation conditions were set as follows: (1) no history of mental illness diagnosis; (2) no psychological treatment in the past 6 months. To address social desirability bias, an anonymous filling and reverse scoring item confusion strategy was adopted, and the Lie scale was introduced to screen for false responses (12 questionnaires with Lie scores > 5 were eliminated). All participants signed an electronic informed consent form.

In this study, “positive items” refer to items with an SCL-90 score of 2 or higher, indicating the presence of some psychological symptoms; “negative items” refer to items with a score of 1 or lower, indicating the absence of significant symptoms. A decrease in the number of positive items indicates a decrease in symptom frequency, while an increase in the number of negative items indicates improved psychological well-being. To ensure consistent understanding, we provided standardized instructions to participants before the assessment, clarifying the scoring scale (1 = none, 2 = mild, 3 = moderate, 4 = severe, 5 = severe). We also randomized the items, ensured anonymity, and emphasized the importance of truthful responses to minimize social desirability bias. After physical exercise, the number of positive items decreased from 35.2 ± 6.1 to 15.3 ± 4.8 (t = 38.64, *p* < 0.001), the number of negative items increased from 54.8 ± 7.2 to 74.7 ± 5.3 (t = −29.17, *p* < 0.001), and the General Symptom Index (GSI) significantly decreased (1.82 ± 0.41 vs. 0.79 ± 0.33), confirming an overall improvement in mental health. The mobile information device and data survey methods used in this article are shown in Table 2.

**Table 2:** Mobile information devices.

Item	Details
Mobile information equipment	Android phone
Data sources	Questionnaire star
Programming language	Python

For the valid questionnaire part, this paper summarizes gender, age and monthly salary, as shown in Table 3.

**Table 3:** Summary of survey respondents.

Item	Male	Female
Gender	Number:180	Number:220
Age	18 to 60	18 to 55
Monthly salary	3,000 to 20,000	2,000 to 15,000

In this paper, the 9 subscales of SCL-90 are represented by M1 ~ M9. Then the score table is shown in Table 4.

**Table 4:** SCL-90 evaluation form.

Item	Weight	M1	M2	M3	M4	M5	M6	M7	M8	M9
Age	w1	a1	a2	a3	a4	a5	a6	a7	a8	a9
Gender	w2	b1	b2	b3	b4	b5	b6	b7	b8	b9
Salary	w3	c1	c2	c3	c4	c5	c6	c7	c8	c9

Since the Mental Health Quality Questionnaire is not the highest behavioral test, the item analysis does not take into account the difficulty of the questionnaire. According to the results of the open-ended questionnaire and previous expert demonstrations, 45 items were formulated in each dimension of cognition, emotion, personality and adaptation. Fifteen items were deleted based on expert opinion and feedback from small-scale testing.

First, delete the items whose q is less than 1.00. Because the discrete degree of these items is insufficient, they cannot effectively distinguish the differences of the subjects in the content, which affects the reliability of the test, so they are deleted. Then each item is correlated with the subscale total score and subscale subdimension. Finally, all the items are arranged according to the total score, and the independent sample consistency test is carried out for the high group of more than 27% and the low group of less than 73%, and the items with no significant difference in the T value at the level of 0.05 are deleted.

### 3.3 Experimental results

**Figure 8:**
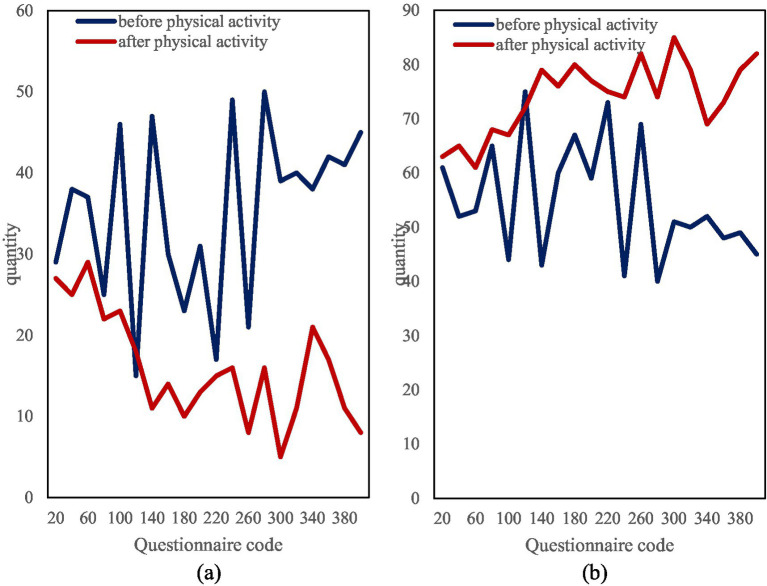
Details of negative and positive numbers. (**a**) Number of positive questions before and after physical activity. (**b**) Number of negative questions before and after exercise physical activity.

In this paper, the weighted sum calculation is performed based on the data recovered from the questionnaire, and the number of positive items and negative items in the SCL-90 scale is obtained, as shown in Figure 8.

It can be seen from the figure that among the 400 questionnaires, the number of positive items before exercise was mostly around 35, and after exercise it dropped to around 15, and the number dropped significantly. Among them, the 140th to 300th investigators had the most significant decline in the number of positive items, the 140th dropped from 47 positive items to only 11, and the 300th dropped from 39 positive items to only 5. In addition, it can also be seen from the negative items that the number of them increased significantly after physical exercise.

**Figure 9:**
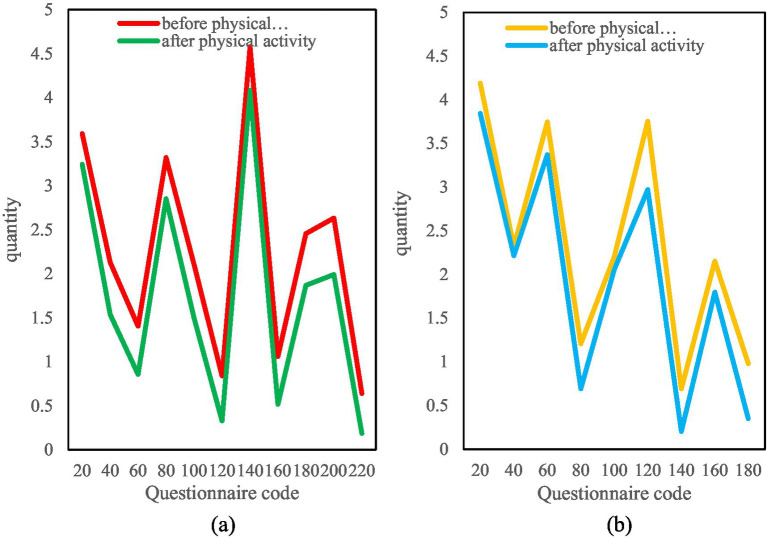
Comparison of gender total symptom index. (**a**) Changes in the index of total symptoms before and after exercise for women. (**b**) Changes in the index of total symptoms before and after exercise in men.

Finally, this paper summarizes the comparison figure of the total symptom index according to gender, age and monthly salary, in which the total symptom index of gender is shown in Figure 9.

It can be seen from Figure 9 that the total symptom index of women before physical exercise is mostly above 2 points, which is more or less psychological symptoms. After physical exercise, the overall symptom index decreased, most of which were below 2 points. From the comparison of men before and after physical exercise, it can be seen that their psychological symptom index has decreased, which also shows that both men and women can relieve psychological symptoms through physical exercise.

**Figure 10:**
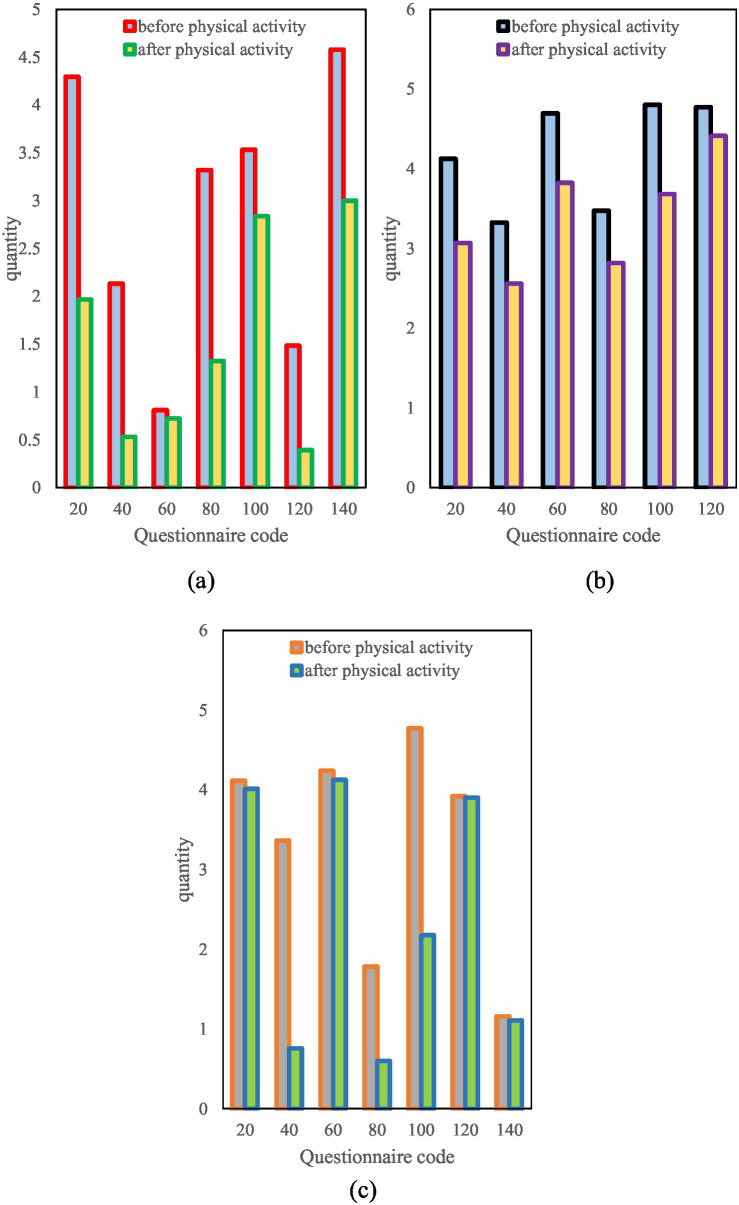
Aggregate symptom index by age. **(a)** Changes in symptom index at 18–30 years old. **(b)** Change in symptom index at 30–50 years old. **(c)** Changes in symptom index over age 50.

In this paper, the surveyed age groups are divided into 18–30 youth group, 30–50 middle-aged group and the elderly group over 50 years old. The symptom index of each group is shown in Figure 10.

As can be seen from Figure 10a, the psychological symptom index of the youth group is quite different, there are more than 4 points and some less than 1 point. However, the overall symptom index decreased significantly after physical exercise. The middle-aged group in Figure 10b has a high psychological symptom index, most of which are above 3.5. The overall index of the middle-aged group decreased after physical exercise, but the decrease was not as obvious as that of the youth group (Figure 10c). The psychological symptom index of the middle-aged and elderly groups is also very different, ranging from a high point of more than 4.5 points to a low point of less than 0.5 points. This difference may stem from the mentality and personal experience of the elderly. After physical exercise in the elderly group, some psychological symptoms decreased a lot, while others hardly changed. This may be because the elderly experienced more things and their mental state was relatively stable. Overall, for young people, physical exercise can significantly relieve psychological stress. For middle-aged people, although physical exercise can relieve some symptoms, the effect is not very obvious. For the elderly, the effect of physical exercise on improving psychological symptoms is quite different, which may be related to the mentality and life experience of the elderly.

**Figure 11:**
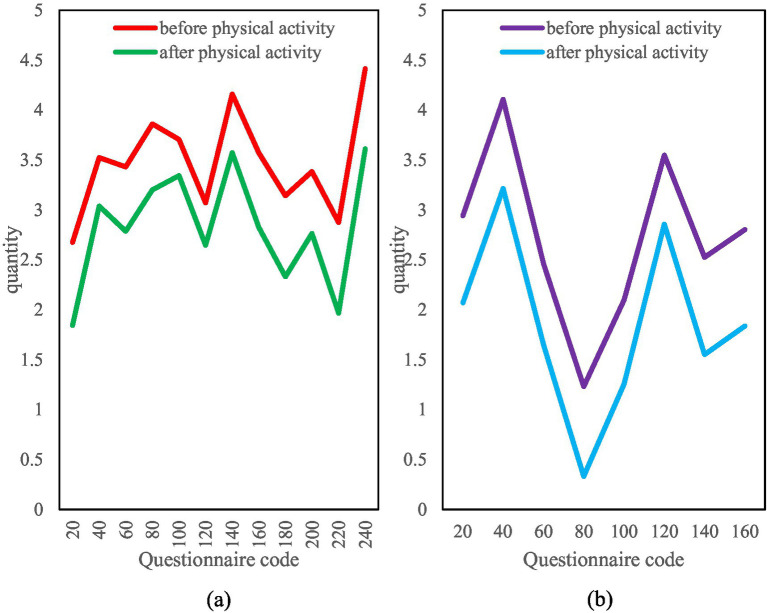
Comparison of psychological symptoms index under different monthly salary. **(a)** Symptom index for monthly salary below 10,000. **(b)** Symptom index for monthly salary above 10,000.

Finally, this paper conducts statistics and comparisons on the psychological symptom index based on the monthly salary of tens of thousands and the monthly salary of less than 10,000, as shown in Figure 11. It can be seen from the figure that for those with a monthly salary of less than 10,000, most of the psychological symptoms index is above 3 points, which are mild to moderate symptoms. The symptom index decreased after physical exercise, but the decrease was not very large. However, the psychological symptom index of the monthly salary of tens of thousands of people has a large gap. Some have less than 1.5 points, and some have more than 4 points. After physical exercise, the overall index has dropped significantly. It shows that for people with considerable monthly income, physical exercise can more effectively promote mental health.

The GSI decrease in the high-income group (≥10,000 yuan) (*Δ* = 1.21 ± 0.19) was significantly greater than that in the low-income group (Δ = 0.83 ± 0.27) (*F* = 7.35, *p* = 0.007). It is important to emphasize that this is a correlation, not a causal relationship, and may reflect the influence of socioeconomic status on access to exercise resources (e.g., differences in high-end gym usage). Further studies are warranted to control for income confounding through quasi-experimental designs.

Regarding the relationship between salary levels and improved mental health, our observational data show that after controlling for variables such as work stress and job satisfaction, employees with higher salary levels showed a more significant trend of improved mental health after the mobile information technology intervention. However, due to the observational design of this study, it is impossible to determine the causal relationship between salary and mental health. This association may reflect a variety of potential mechanisms: higher salaries may provide more resources to cope with work stress; or employees with better mental health may be more likely to receive salary increases; or a third variable (such as organizational status) may affect both salary levels and mental health. Future research should adopt longitudinal designs or quasi-experimental methods to further explore the dynamic relationship between salary changes and improved mental health to clarify its potential causal mechanism.

## 4 Conclusion

This article first introduces the background and significance of research on the effects of physical exercise on mental health, and describes the research methods and experimental results. The introduction summarizes the role of physical activity on mental health and reviews the current state of research on the effects of physical activity on mental health and the role of mobile information technology both domestically and internationally. The theoretical section details physical exercise programs and their benefits, mental health assessment criteria, the causes and treatments of psychological problems, and introduces the principles of the weighted summation algorithm. The experimental section describes in detail the questionnaire design process and data analysis methods. Statistical results show that exercise significantly reduces positive items and significantly increases negative items on the SCL-90. The overall psychological symptom index decreased in both men and women and across different age groups, but the extent of the decrease varied. The symptom index decreased less significantly in low- and middle-income groups, while the improvement was greater in high-income groups. Overall, physical exercise promotes mental health across age, gender, and income levels, with particularly significant effects among young people and those with high incomes.

## Data Availability

The original contributions presented in the study are included in the article/supplementary material, further inquiries can be directed to the corresponding author.
